# Extracellular vesicles: cargo loading, degradation and secretory pathways, and their intersection with autophagy

**DOI:** 10.20517/evcna.2025.21

**Published:** 2025-07-16

**Authors:** Jinzhe Ju, Sophie M.L. Neuen, Marc van Zandvoort, Tom G.H. Keulers, Kasper M.A. Rouschop

**Affiliations:** ^1^Department of Radiation Oncology (Maastro), GROW - School for Oncology and Reproduction, Maastricht University Medical Center+, Maastricht 6229 HX, The Netherlands.; ^2^Department of Pediatrics, GROW - School for Oncology and Reproduction, Maastricht University Medical Center+, MosaKids Children’s Hospital, Maastricht 6229 ER, The Netherlands.; ^3^Department of Genetics and Cell Biology, Cardiovascular Research Institute Maastricht (CARIM), GROW - School for Oncology and Reproduction, Maastricht University, Maastricht 6229 ER, The Netherlands.; ^4^Institute for Molecular and Cardiovascular Research (IMCAR), University Hospital Aachen, Aachen 52074, Germany.

**Keywords:** Extracellular vesicle, autophagy, ESCRT, biogenesis, lysosome

## Abstract

Extracellular vesicles (EVs) are secreted by nearly all cell types and fulfil a crucial role in intercellular communication by transporting diverse cargo, including enzymes, mRNA, growth factors, chemokines, and cytokines. Although EVs were initially thought to primarily function in waste elimination, it is now clear that they can be diverted from degradation and instead actively secreted to mediate intercellular communication. While the processes of EV biogenesis, degradation, and release have been extensively studied, many aspects remain poorly understood. The involvement of molecular pathways shared by EV biogenesis and autophagy - a lysosome-mediated disposal mechanism - suggests the existence of common regulatory controls. Despite the partial overlap in molecular machineries involved in cargo sorting, the mechanisms that balance the degradation and secretory pathways of EVs, as well as their interplay with autophagy, remain elusive. This review discusses the molecular machinery that dictates the selective cargo loading into EVs. Additionally, it examines the coordination between degradation and secretory pathways in EV biology and situates these processes within the broader context of autophagy. The substantial overlap in molecular pathways, shared proteins, and complementary mechanisms suggests a high degree of coordination between these systems.

## INTRODUCTION

Intercellular signaling is a vital biological process essential to maintain homeostasis, support growth, and enable responses to damage and stressors. Intercellular communication can be achieved by direct cell-to-cell contact, the secretion of signaling molecules, or the intercellular trafficking of extracellular vesicles (EVs)^[1]^. After their initial discovery^[2]^, EVs have been detected in all biological fluids and in conditioned media of cultured cells. EVs are secreted from almost every cell type, mediating intercellular communication through the transport of diverse cargo, including enzymes, mRNAs, microRNAs (miRNAs), growth factors, chemokines, and cytokines^[3,4]^. Due to their capacity to mediate intercellular communication, EVs have been investigated in both physiological and pathophysiological processes, including cancer and neurodegenerative disorders^[1,3]^. The role of EVs in both physiological and pathophysiological processes contributes to the great potential of EVs as diagnostic tools and/or therapeutic targets. As such, the clinical and scientific interest in the field of EVs has increased over the last decades.

**Figure 1:**
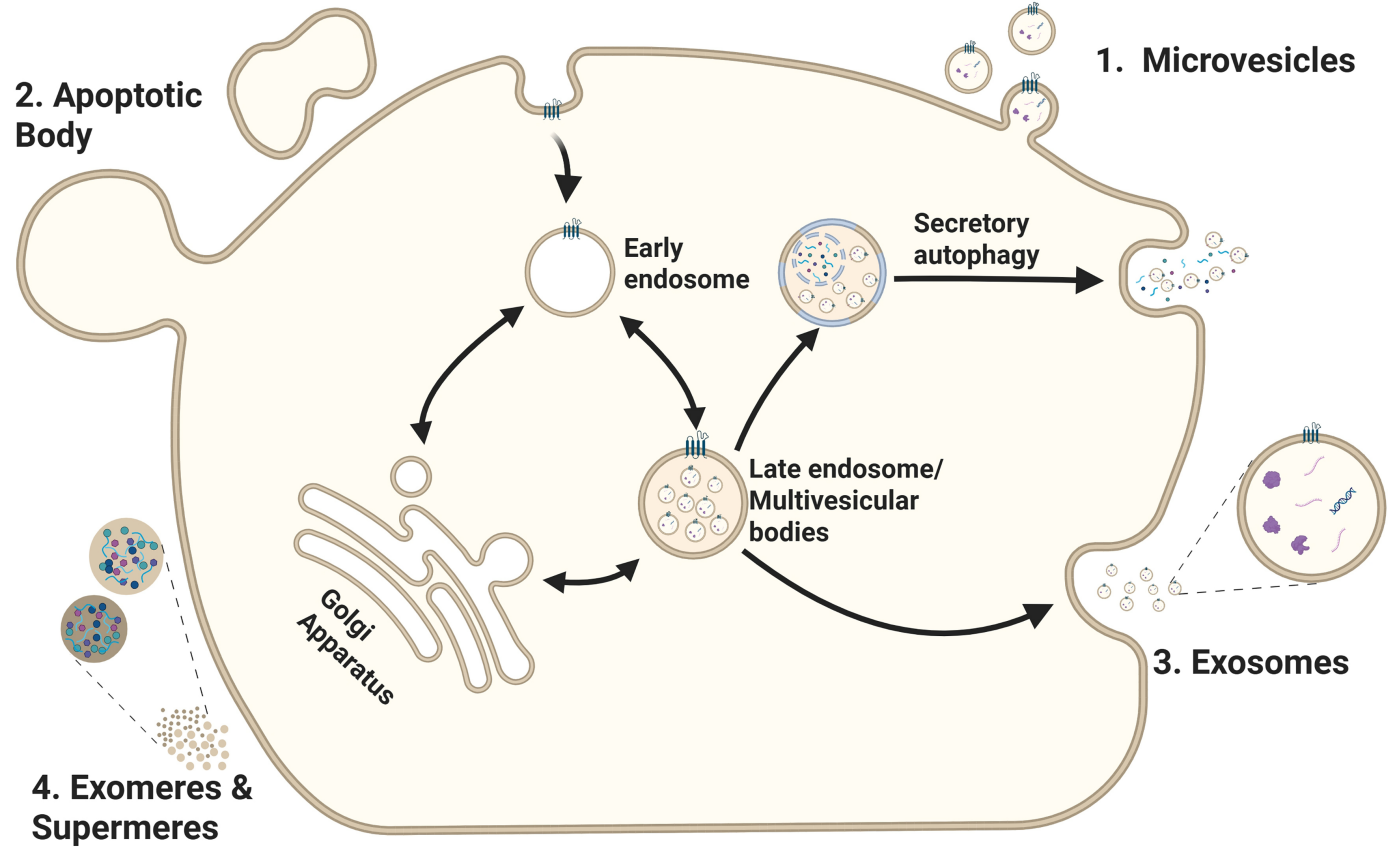
EV biogenesis and secretion. This scheme illustrates the routes involved in the formation and release of distinct EV subtypes: (1) Microvesicles are generated by outward budding and fission of the plasma membrane; (2) Apoptotic bodies are created during apoptosis via plasma membrane blebbing; (3) Exosomes originate from the inward budding of the early endosome membrane, forming ILVs that mature into MVBs. Upon fusion of the MVB with the plasma membrane, ILVs are released into the extracellular space as exosomes. Alternatively, the MVBs can fuse with autophagosomes. Cargo can then be released through secretory autophagy and bypasses lysosomal degradation; (4) Exomeres and supermeres are smaller nanoparticles released through currently undefined mechanisms. Created in BioRender. Ju, J. (2025) https://BioRender.com/ddkj6wp. EV: Extracellular vesicle; ILVs: intraluminal vesicles; MVBs: multivesicular bodies.

Based on the mechanism of biogenesis and secretion, EVs are categorized as microvesicles, apoptotic bodies (ApoBDs), exosomes, (large) oncosomes, and migrasomes. In addition, non-vesicular extracellular nanoparticles such as exomeres and supermeres have been identified, which lack a lipid bilayer and are considered distinct from classical EVs^[3,5]^. Microvesicles, also known as ectosomes, are between 100 nm and 1-2 μm in diameter and are generated by pinching off the plasma membrane^[3,5]^ [Figure 1 and Table 1]. (Large) oncosomes are an atypically large (1-10 μm in diameter) subtype of microvesicles derived from cancer cells with distinct cargo and associated with advanced disease^[6,7]^. With a diameter between 50 nm and 5 μm, ApoBDs display the largest range of EV sizes^[5]^ [Table 1]. In contrast to the other types of EVs, ApoBDs are produced during apoptosis by blebbing off the plasma membrane of apoptotic cells. An increased number of ApoBDs is usually observed in tumors because macrophages fail to remove apoptotic cells in time^[8]^. ApoBDs display diverse signaling properties^[9,10]^ that may influence disease progression [Figure 1]^[9-11]^. Exosomes originate from multivesicular bodies (MVBs) derived from the endosomal pathway and are released into the extracellular environment when MVBs fuse with the plasma membrane^[12]^. The diameter of exosomes ranges from 30 to 150 nm, attributing them to one of the smallest types of EVs^[5,13]^ [Table 1]. Migrasomes are large vesicular structures (up to 3 μm in diameter) that form at the tips or intersections of retraction fibers during cell migration. These structures contain numerous smaller vesicles and are involved in a process termed “migracytosis”, facilitating the release of cytoplasmic contents into the extracellular environment^[14,15]^. Finally, exomeres and supermeres are non-membranous extracellular nanoparticles, typically isolated from EV-depleted medium following centrifugation at 196,000 *g* for 16 h and 367,000 *g* for 16 h, respectively. These nanoparticles are slightly smaller than EVs, with sizes averaging around 35 nm for exomeres and 29 nm for supermeres. However, their secretion mechanisms remain largely unknown^[16,17]^.

**Table 1:** Characteristics to distinguish between different types of extracellular vesicles

**Characteristics**	**Exosomes**	**Microvesicles**
Biogenesis	Endomembrane system	Plasma membrane
Size	30-150 nm	100 nm-2 μm
Shape	Heterogeneous	Heterogeneous
Sedimentation	~100,000 × *g*	~10,000 × *g*
Known enriched proteins	- ESCRT and accessory proteins - Tetraspanins (CD9, CD63 and CD81) - Annexin - Flotillins	- Tetraspanins - Heat shock proteins - Integrins - TSG101 - CD274
Known lipids	- Cholesterol - Phospholipids - Sphingolipids - Ceramide - Glycerolipids - Diglyceride - Triglyceride	- Cholesterol - Phospholipids - Triglyceride
Known nucleic acids	- ncRNA - mRNA - miRNA - DNA	- ncRNA - mRNA - miRNA - DNA

Data obtained from ExoCarta and Vesiclepedia^[18,19]^. ESCRT: Endosomal sorting complex required for transport; TSG101: tumor susceptibility gene 101.

EVs carry a distinguishable molecular cargo, which also aids in their classification [Table 1]. To qualify EV cargo, proteomic-, lipidomic-, and transcriptomic analyses have been conducted and assembled into several online databases^[18,19]^. Despite these categorizations, the exact mechanisms of cargo sorting into EVs remain largely unclear, and only a few selective mechanisms of cargo segregation have been established. Similar to EV biogenesis, intracellular degradation pathways such as macroautophagy, microautophagy, chaperone-mediated autophagy (CMA), and endosomal degradation also involve the recruitment of cargo into vesicular compartments. However, unlike these degradation pathways, EV-associated cargo is initially sorted for secretion, but can still be subjected to lysosomal degradation, dependent on cellular conditions^[12]^. The subsequent secretion of EVs with their cargo enables intercellular communication^[20,21]^. Although discussed regularly, the coordination of the degradation and secretory pathways of EVs is still poorly understood^[22-26]^. To advance our understanding of the EV field, several key questions need to be addressed: How is cargo selectively loaded, and what mechanisms regulate the segregation of cargo? What determines whether EVs are directed toward the degradation or secretory pathway?

As EVs can originate from endosomal precursors, a comprehensive and systematic approach for studying endosomal trafficking is essential to uncover how endosomes are directed toward their distinct fates - degradation, secretion, or recycling.

Autophagy is a mechanism for the degradation of intracellular vesicles (autophagosomes) that encapsulate cytosolic proteins and organelles. Fusion of autophagosomes with lysosomes enables degradation of cargo. Autophagy is considered essential for homeostasis, development, and the prevention of diverse diseases and infections^[26]^. Three different types of autophagy exist: macroautophagy, CMA, and microautophagy, which differ in terms of signaling pathways, cargo segregation, and the mechanism of delivery to lysosomes^[27]^. Several studies have demonstrated that the interlinked processes of EV- and autophagy-related actions facilitate homeostasis through both degradation and secretory pathways in both physiological and pathophysiological conditions^[12,23,28,29]^. Nevertheless, to what extent the different mechanisms of autophagy and EV biogenesis overlap or are even cross-regulated remains to be investigated. This literature review aims to elucidate cargo sorting into EVs and the regulation of the balance between the degradation and secretory pathways.

## BIOGENESIS AND THE SECRETION OF EVs

An single cell can secrete multiple types of EVs. The mutual properties and the distinct features observed after database comparisons (restrictions: Homo Sapiens AND Exosome OR microvesicle AND isolation method Differential/ultracentrifugation OR size exclusion chromatography AND minimal 3 times identified) are presented in Table 1. These data are in line with large mass spectrometry analyses on exosomes and EVs. As ApoBDs are primarily a product of programmed cell death rather than mediators of intercellular communication mechanism and the mechanism of migrasome biogenesis is currently unknown, this review will focus solely on exosomes and microvesicles.

### Cargo sorting into EVs

#### Exosomes

The biogenesis of exosomes is a complex biological process involving multiple cellular pathways that regulate their composition, sorted proteins, and other loaded cargo^[5]^. The biogenesis of exosomes is closely linked to the endolysosomal system. Early, or sorting endosomes, receive input from the plasma membrane through endocytosis. During their maturation, the integration of vesicles from the trans-Golgi network (TGN) contributes to the diversification and expansion of the endosomal network, enhancing its capacity for recycling, sorting, or degradation^[30]^. The sorted molecules are either recycled for reuse at the plasma membrane via Ras-related protein (RAB)11-positive recycling endosomes (RE) or are trafficked to the TGN via the retromer complex or to the lysosome^[31,32]^. Classically, the molecules destined for further processing are sorted into luminal invaginations of the early endosomal membrane, which bud inward to form intraluminal vesicles (ILVs). These ILVs accumulate within the lumen of maturing endosomes, which develop into MVBs. The maturation of early endosomes into MVBs is driven by the replacement of RAB5 with RAB7. Like the early endosome, MVBs are involved in cargo sorting and intracellular trafficking^[4,31]^. The content of MVBs can either be released into the extracellular environment via fusion with the plasma membrane - resulting in the release of exosomes - or targeted for degradation via fusion with lysosomes^[4]^ [Figure 1].

**Figure 2:**
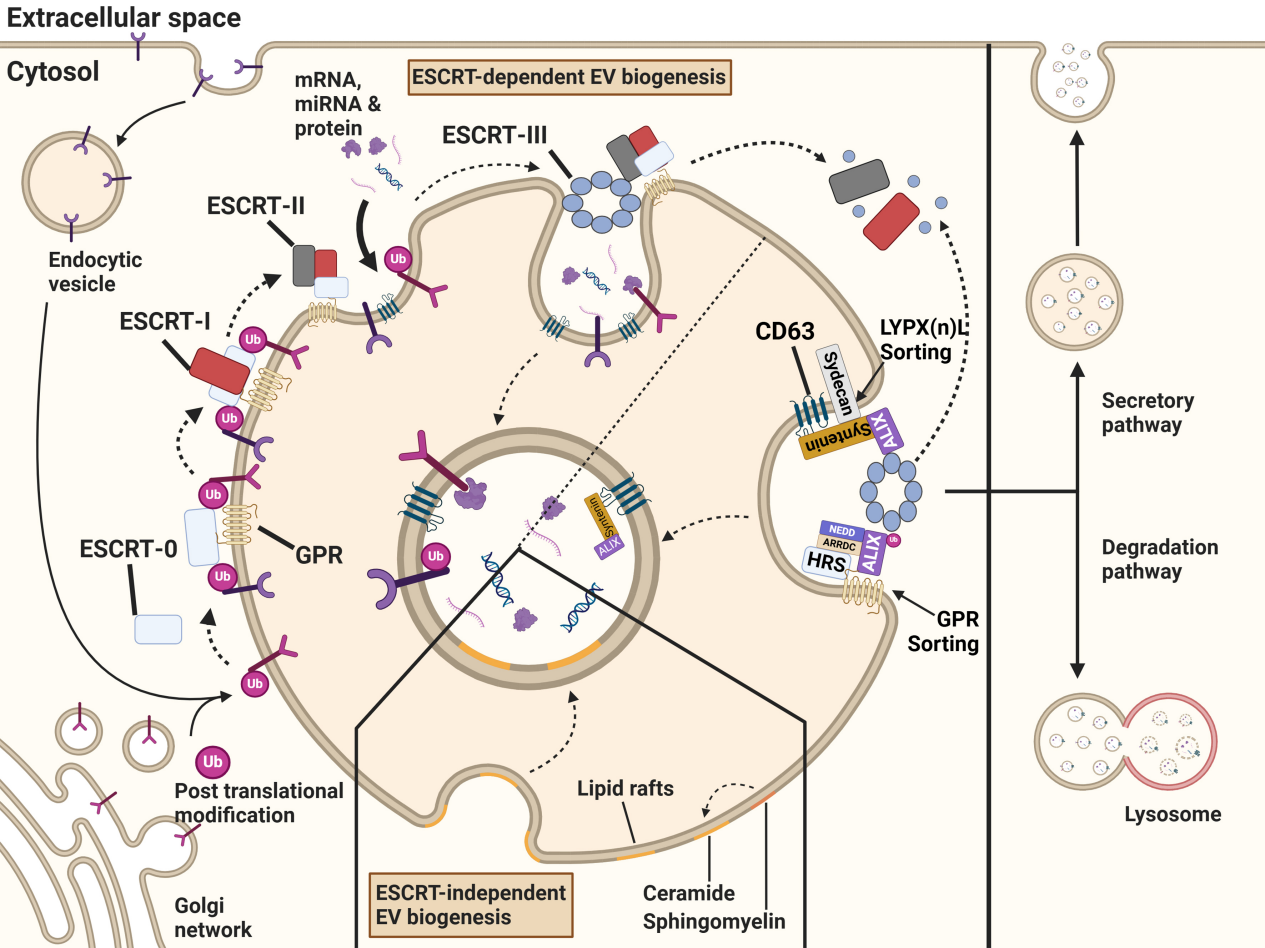
Biogenesis, degradation and secretion of exosomes. Exosomal cargo is recruited by the ESCRT-dependent or ESCRT-independent pathways. Endocytosed proteins or post-translationally modified proteins that originate from the trans-Golgi network and are destined for the endosome, are recruited to emerging exosomes. The ESCRT-0 complex recognizes the post-translational modification and sorts them into microdomains. ESCRT-0 correlates with ESCRT-I, followed by the recruitment of ESCRT-II. The complexes initiate the invagination of the endosomal membrane and formation of ILVs within MVBs. The ESCRT-III subunit is recruited to the endosome and leads to the scission of the vesicles. Due to the binding of ESCRT-III to accessory proteins, free ubiquitin molecules and the ESCRT machinery are recycled. Through lipid raft microdomains enriched in sphingolipids and tetraspanins, cargo can be sorted into ILVs independently of the ESCRT machinery. These ILVs are either secreted as exosomes or degraded following lysosomal fusion. Created in BioRender. Ju, J. (2025) https://BioRender.com/75gp5la. ESCRT: Endosomal sorting complex required for transport; ILVs: intraluminal vesicles; MVBs: multivesicular bodies; GPR: G protein-coupled receptor.

Although the exact mechanism of biogenesis is not fully understood, different mechanisms involved in cargo sorting have been identified. Commonly, the pathways for cargo sorting during the biogenesis of exosomes are divided into the endosomal sorting complex required for transport (ESCRT)-dependent pathways and ESCRT-independent pathways [Figure 2].

ESCRT-dependent cargo sorting The ESCRT machinery is organized into four complexes, ESCRT-0, ESCRT-I, ESCRT-II, and ESCRT-III, which are coupled to accessory proteins. In an intricate and coordinated process, ubiquitinated cargo is loaded into the endosomes, and thereby the ESCRT machinery regulates the formation of ILVs^[33]^. Before the recruitment of the ESCRT machinery, tetraspanins are enriched in the endosomal membrane and play a key role in early cargo sorting, microdomain organization, and ultimately ESCRT-independent ILV formation. They serve as an important preparatory step for the downstream processes of MVB biogenesis^[34]^.

The first component of the ESCRT machinery to localize at the endosome is the ESCRT-0 complex, which binds to phosphatidylinositol 3-phosphate [PI(3)P]. Once recruited to the endosomes, the ESCRT-0 complex initiates cargo sorting by recognizing and binding to the ubiquitin tags on membrane proteins^[35]^ [Figure 2]. Within this complex, several proteins play crucial roles in cargo selection. Notably, hepatocyte growth factor-regulated tyrosine kinase substrate (HRS) facilitates the recognition of ubiquitinated cargo. HRS interacts with clathrin, signal-transducing adapter molecule (STAM1), and EPS15 to form a complex essential for efficient cargo sorting and processing^[36]^.

Binding of adaptor proteins to ubiquitin ligases (E3) further determines the specificity of cargo sorting. For example, neural precursor cells-expressed developmentally downregulated protein 4 (NEDD4), through a process called NEDDylation^[37]^, interacts with arrestin domain-containing proteins (ARRDCs)^[38]^. ARRDC3, in particular, binds to HRS and promotes the ubiquitination of ALG2-interacting protein X (ALIX), thereby facilitating G protein-coupled receptor (GPR) sorting and the recruitment of ESCRT-III components^[39]^. Additionally, GPR143 mediates the recruitment of HRS to facilitate the sorting of proteins such as the epidermal growth factor receptor (EGFR) into ILVs^[40]^. Consistently, proteomic analyses have demonstrated that knockout of ARRDC1 alters the protein cargo of exosomes^[41]^. Although ARDDC1 plays a prominent role in exosomal cargo sorting, it also regulates microvesicle budding through interactions with the tumor susceptibility gene 101 (TSG101) and NEDD4-ubiquitinated ALIX^[39,42]^ [Figure 2]. Moreover, another family member, ARRDC4, controls the release of divalent metal transporter 1 (DMT1), a major iron transporter, via K-29-linked ubiquitination^[38]^. Intriguingly, other ubiquitin ligases, such as the CUL1-RING ubiquitin ligase (CRL1), determine the fate of endolysosomes by recruiting factor S-phase kinase-associated protein 1 (SKP1). In its phosphorylated state, SKP1 enhances endolysosomal degradation, whereas in its SUMOylated state, it promotes secretion^[43]^.

Hence, other than ubiquitination and NEDDylation, several post-translational modifications, including SUMOylation, ISGylation, phosphorylation, and glycosylation, also control cargo sorting. SUMOylation influences cargo sorting through interaction with phosphoinositols in the ESCRT machinery. For example, SUMOylation of Synuclein (SNCA), a neurotoxic protein in Parkinson’s disease, promotes its recruitment to EVs^[44]^. The addition of the ubiquitin-like modifier 15 (ISG15) facilitates cargo sorting by aggregating and degrading TSG101 subunits^[45]^. Additionally, clathrin supports cargo sorting by concentrating the ESCRT machinery within confined microdomains and locally generating a high ESCRT gradient^[46]^, thereby facilitating the orderly recruitment of ESCRT-0^[36]^.

Proteomic analyses indicate that phosphorylated proteins are enriched in EVs, suggesting that phosphorylation is required for efficient cargo sorting^[34]^. In line with this, Liem *et al.* demonstrated that, after PI3K/AKT pathway activation, phosphorylated AKT (but not in its native form) is selectively targeted to EVs^[47]^. Intriguingly, phosphorylated AKT promotes phosphorylation of nuclear factor kappa-light-chain-enhancer of activated B cells (NF-κB), thereby enhancing the transcription of genes involved in ESCRT-dependent and -independent sorting pathways, ultimately leading to increased EV release^[48]^. These results suggest that phosphorylation events are key in cargo recruitment and EV biogenesis. Nevertheless, the high abundancy of phosphorylated proteins cannot be solely ascribed to a selective cargo sorting mechanism; rather, it indicates that sorting is the net result of complex interactions^[37]^.

Glycosylation, another type of post-translational modification, is also associated with cargo sorting. N-glycans include high-mannose type modifications as well as complex types composed of additional monosaccharides such as polyLacNac^[49]^. Both types are enriched in EVs derived from various cell types. For example, high-mannose-modified glycoproteins can bind to galectins and the vesicular-integral membrane protein of 36 kDa (VIP36). The subsequent oligomerization of these glycoproteins facilitates their sorting into vesicles^[34]^.

The interaction between HRS and TSG101 leads to ESCRT-I recruitment. Comparable to ESCRT-0, ESCRT-I binds to ubiquitin moieties and mediates cargo sorting^[34,44]^. Furthermore, the association of TSG101 with Ras GTPase-activating-like protein IQGAP1 and gasdermin D (GSDMD) in inflammasome-activated intestinal epithelial cells results in selective IL‐1β sorting, highlighting its importance in cargo recruitment^[50]^. Additionally, Bänfer et al. identified a highly conserved tetrapeptide P(S/T)AP late domain motif in the cytoplasmic tail of E-cadherin, which mediates its interaction with TSG101 and is essential for E-cadherin recruitment into EVs^[51]^. Whether other proteins contain similar tetrapeptides that enable selective sorting remains to be elucidated.

Following the interaction of ESCRT-0 with ESCRT-I, ESCRT-II is recruited and initiates the oligomerization of ESCRT-III. Next, ESCRT-I starts the invagination of the endosomal membrane to shape ILVs that engulf cytosolic proteins, a process further supported by ESCRT-II^[34,52]^. The association of ESCRT-III with accessory proteins triggers membrane fission, leading to the release of the ESCRT machinery from the endosomal membrane and enabling its recycling^[34,35]^ [Figure 2]. Alternatively, exosomes can also originate from RAB11-positive RE, which are distinct from those formed in late endosomal MVBs^[53]^. In this context, ESCRT‐III proteins selectively regulate the cargo loading of RAB11‐ and RAB11a‐exosomes independently of ubiquitinylation/deubiquitinylation^[54]^. The accessory protein ALIX recruits ESCRT-III to late endosomes independently of ESCRT-II^[55]^. This process relies on the interaction between ALIX and lysobisphosphatidic acid (LBPA) in the BRO1 domain. Additionally, ALIX- and ESCRT-III interaction facilitates the sorting and delivery of tetraspanins (TSPANs) into exosomes. As a result, ALIX is co-secreted with TSPANs and exhibits both ubiquitin-dependent and ubiquitin-independent binding capacities^[55]^. The multifunctional enzyme transglutaminase type 2 (TG2) interacts not only with ALIX but also with TSG101, thereby regulating cargo selection under cellular stress conditions^[56]^. Interestingly, TG2 also interacts with NEDD4 and GRP75^[57,58]^.

Furthermore, ubiquitously expressed TSPANs such as CD9, CD81, and CD63 are actively sorted into EVs^[5,59,60]^. Intriguingly, CD63 is recruited by syntenin-1^[55,59]^, a process dependent on the syndecan-syntenin-ALIX complex (SSA)^[34,61]^. Syndecan heparan sulfate proteoglycans and the adaptor protein syntenin-1 interact with ALIX via three LYPX(n)L motifs at its N-terminus, and with syndecans through their PDZ domains. ALIX, in turn, associates with components of the ESCRT machinery at the MVB, facilitating the loading of syntenin-syndecan-sorted cargo into exosomes. Intracellular cleavage of syndecans by heparanase stimulates the sorting of syntenin-1, CD63, and other exosomal cargo, whereas the sorting of CD81 and flotillin-1 remains unaffected^[61]^.

ESCRT-independent cargo sorting Despite the critical role of the ESCRT machinery in MVB formation and exosome biogenesis, cells deficient in components of ESCRT complexes are still capable of generating MVBs, suggesting the presence of alternative sorting mechanisms. Among these, lipid rafts have emerged as key players in cargo recruitment and vesicle formation. Lipid rafts are specialized microdomains enriched in sphingolipids and TSPANs, which are pivotal for exosomal cargo sorting and membrane dynamics [Figure 2].

One alternative mechanism is the enzymatic conversion of sphingomyelin to ceramide by sphingomyelinase (nSMase). This lipid remodeling facilitates the formation of ceramide-enriched microdomains that enable sorting of specific miRNAs^[34,62]^. In addition to ceramide-enriched microdomains, ceramide derivatives also contribute to exosomal cargo sorting. For instance, ceramidase hydrolyzes ceramide into sphingosine, which is subsequently phosphorylated by sphingosine kinase (SsphK), forming sphingosine 1-phosphate (S1P), a potent signaling lipid^[63]^ [Figure 2]. The bioactive form S1P activates its cognate receptor, the G protein-coupled S1P receptor, triggering dissociation of the G_βγ_ subunit from the G_i_ subunit^[63]^.

**Figure 3:**
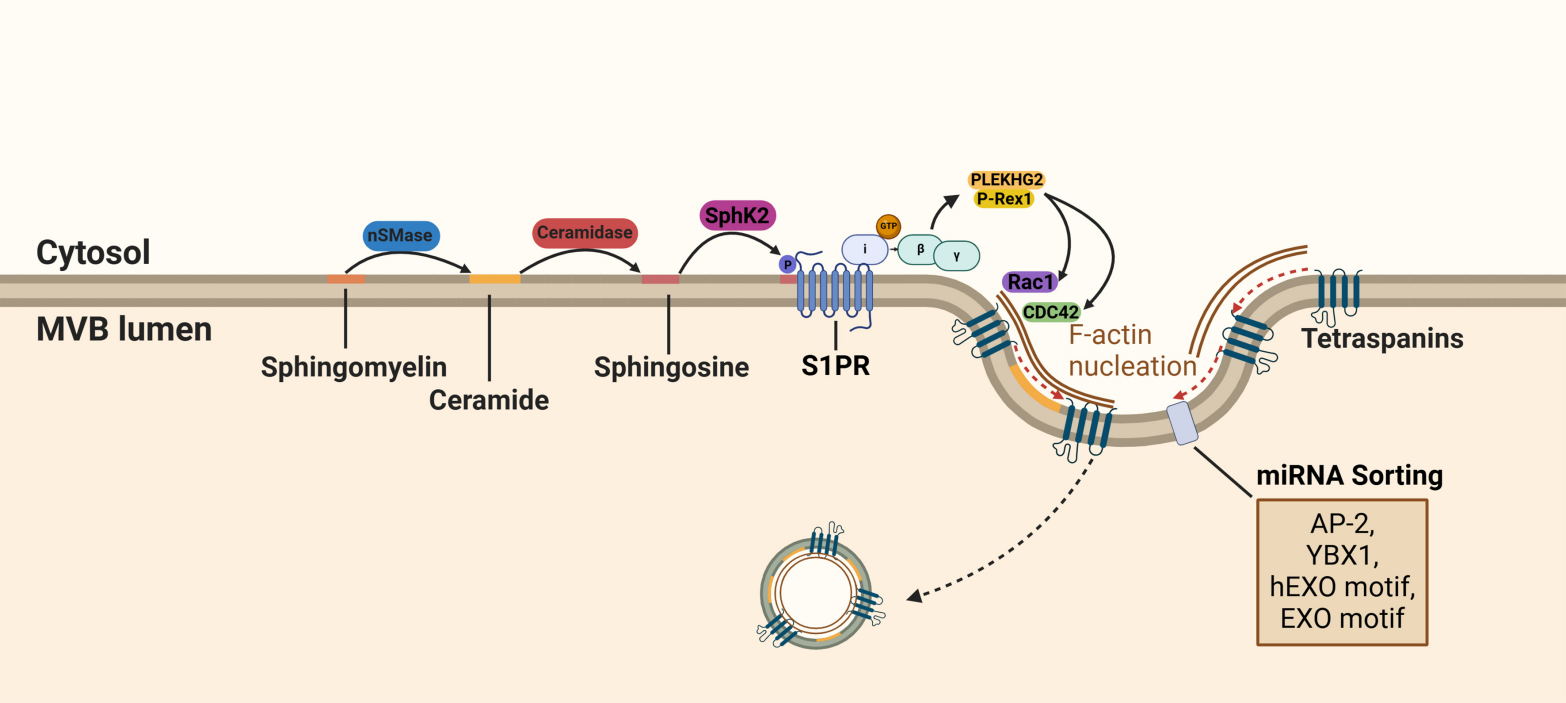
S1P receptor activation in ILV cargo sorting. During the maturation of MVBs, sphingolipids are hydrolyzed into ceramide and S1P. Activation of the S1P receptor on MVBs results in the dissociation of the G_βγ_ subunit from the G_i_ subunit and stimulates interaction with PLEKHG2 and P-Rex1. The subsequent activation of components of the Rho family GTPases (CDC42 and RAC1) results in F-actin alignment. F-actin is required for cargo sorting into exosomes through its adaptor protein function. Created in BioRender. Ju, J. (2025) https://BioRender.com/vhrj9op. S1P: Sphingosine 1-phosphotase; ILVs: intraluminal vesicles; MVBs: multivesicular bodies; RAC1: Ras-related C3 botulinum toxin substrate 1; EXO: a common short sequence motif enriched in miRNA; AP-2: adaptor-related protein complex 2; YBX1: Y-box protein I; CDC42: cell division control protein 42 homolog; SphK2: sphingosine kinase 2.

The dissociated G_βγ_ subunit initiates the interaction of PLEKHG2 and P-Rex1 and activates members of the Rho family of GTPases^[64,65]^ [Figure 3]. Kajimoto *et al.* showed that the activation of the Rho family GTPases cell division cycle 42 (CDC42) and Ras-related C3 botulinum toxin substrate 1 (RAC1) is responsible for the nucleation of actin filament (F-actin). Inhibition of the G_βγ_ subunit inhibits F-actin nucleation and reduces cargo trafficking into exosomes^[65]^ [Figure 3].

Moreover, active CDC42, a plasma membrane-associated small GTPase, mediates the interaction between GSDMD and IQ motif-containing GTPase-activating protein 1 (IQGAP1). This complex functions as an adaptor protein for the ESCRT-I protein TSG101-mediated sorting, regulating the exosomal release of interleukin-1β (IL-1β)^[50]^.

Intriguingly, in response to elevated intracellular Ca^2+^, GSDMD-IQGAP1-CDC42 complexation is disrupted by the calcium‐responsive protein calmodulin, which competitively binds to IQGAP1^[66,67]^. This inhibition reduces the loading of ubiquitinated IL‐1β into EVs^[50]^. Whether and how CDC42 adapts ubiquitinated protein sorting within the ESCRT-dependent machinery or how CDC42 modulates both ESCRT-dependent and -independent mechanisms requires further investigation.

The sorting of cargo into exosomes is regulated by RAB GTPases and TSPANs, which control the fate of the MVBs by directing them either to the plasma membrane or to the lysosome for degradation. For instance, RAB27A and RAB27B contribute to the biogenesis of exosomes by recruiting MVBs to the plasma membrane^[34]^. RAB4A, which localizes on the early endosome, contributes to cargo sorting through the formation of an endosomal complex with adaptor protein-3 (AP-3), the motor protein Kinesin-like protein (KIF3), and rabenosyn-5 (RBSN)^[68]^. Interestingly, RAB11 and RAB7 are involved in mRNA expression of proteins required for ESCRT-dependent and -independent sorting, respectively, through the phosphorylation of NF-κβ^[48]^. This highlights the diverse roles of RAB GTPases in cargo sorting in exosomes.

#### Microvesicles

**Figure 4:**
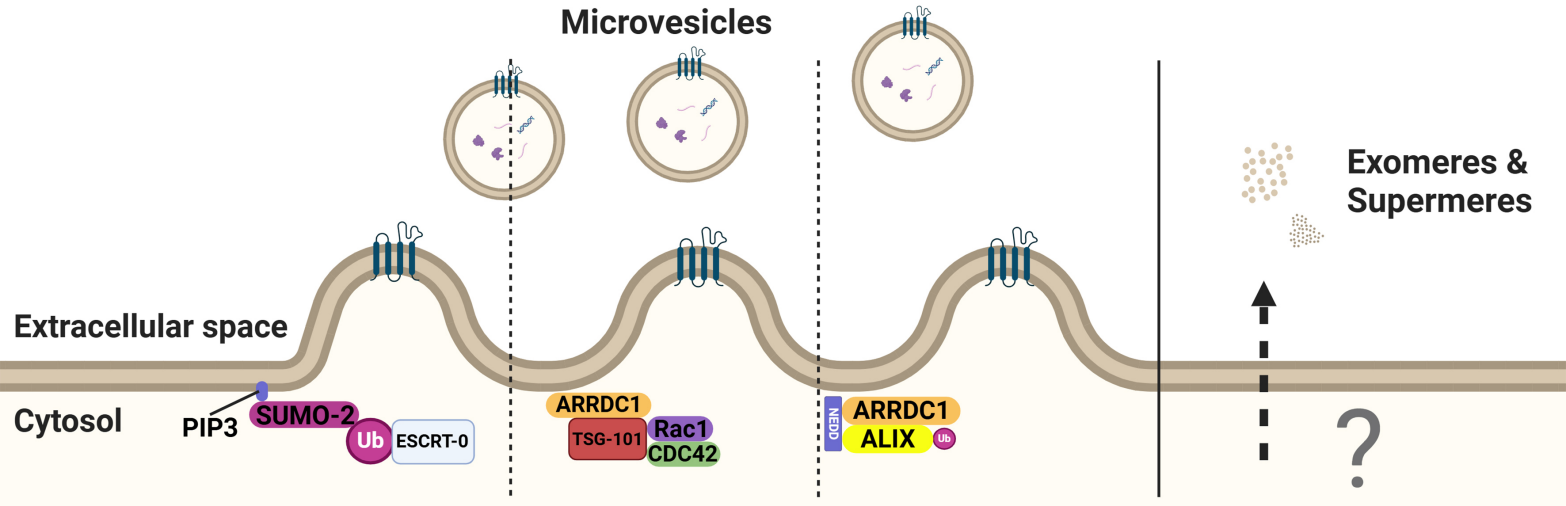
PIP3 mediates SUMO anchoring and the subsequent ubiquitination of ESCRT-0. ARRDC1 regulates the production of microvesicles by binding to TSG101 and facilitating ubiquitination of ALIX. The release mechanisms of exomeres and supermeres remain unknown. Created in BioRender. Ju, J. (2025) https://BioRender.com/vxlt8r5. PIP3: Phosphatidylinositol (3,4,5)-trisphosphate; SUMO: small ubiquitin-related modifier; ESCRT: endosomal sorting complex required for transport; ALIX: ALG2-interacting protein X; ARRDC1: arrestin domain-containing protein 1; Rac1: Ras-related C3 botulinum toxin substrate 1; CDC42: cell division control protein 42 homolog.

Exosomes and microvesicles are two primary types of EVs that are released by normal cells under physiological conditions^[69]^. The biogenesis of microvesicles is mediated by direct outward budding of the plasma membrane, which is followed by their release via pinching off of the plasma membrane. Through recruitment of the TSG101 subunit of the ESCRT machinery to the plasma membrane via binding to Arrestin 1 domain-containing protein 1 (ARRDC1), ESCRT complexes are recruited to microvesicles^[70,71]^. Consistently, silencing ARRDC1 alters the protein cargo in microvesicles^[41]^. Although mechanistically poorly understood, attachment of SUMO-2 to the plasma membrane through phosphatidylinositol (3,4,5)-trisphosphate (PIP_3_) for the initiation of shedding into a vesicle is thought to contribute to cargo sorting^[44]^ [Figure 4].

In certain cell lines, plasma membrane budding can be more efficient than MVB (exosomal) pathways. For example, in HEK293F cells, directing CD63 to the plasma membrane significantly increased its incorporation into secreted EV, while mislocalization of CD9 to endosomal compartments reduces its secretion efficiency^[72]^. Furthermore, although ALIX, RAB27A, and CD63 are commonly associated with exosome and microvesicle biogenesis or release, their silencing does not significantly affect overall EV secretion in this context, thereby challenging the traditional view that EV release is predominantly endosome-dependent^[72]^. Furthermore, Ai et al. showed that Syntenin drives EV-dependent CD63 secretion by blocking its endocytosis and loading into CD81/CD9 EVs. High expression of CD63 results in the inhibition of its own endocytosis, leading to its accumulation at the plasma membrane and enhancing EV secretion^[73]^. Moreover, the absence of ALIX showed no effect on EV release, supporting the notion that Syntenin-induced EV biogenesis is an ALIX-independent process^[72]^.

Intriguingly, Brain-specific angiogenesis inhibitor 1-associated protein 2 (BAIAP2) interacts with RAC1 via its I-BAR domain (I-BAR senses curvature and induces negative membrane curvatures at the plasma membrane). Both RAC1 and CDC42 bind to TSG101, but the secretion of BAIAP2+ EVs is independent of TSG101^[50,65,74]^. Nevertheless, only half of BAIAP2+ EVs contain CD81 and the vesicular release of CD81 is not affected by BAIAP2 deficiency. This suggests that two distinct types of plasma membrane-derived EVs exist^[74]^. Unfortunately, there is no standard established to classify the different types of vesicles after secretion, and many studies focus only on exosomes^[70]^. Due to this bias, the literature focusing on microvesicles is limited and the mechanism of the segregation of cargo that is loaded into microvesicles is less defined compared to exosomes.

#### Exomeres and supermeres

Exomeres are non-membranous extracellular nanoparticles (~35 nm) that were first identified and isolated through asymmetric flow field-flow fractionation (AF4). Exomeres display unique N-glycosylation, protein, lipid, DNA and RNA profiles, and biophysical properties^[16]^. Zhang *et al.* showed the potent signaling and growth-promoting activities through β-galactoside α2,6-sialyltransferase 1 (ST6Gal-I) that adds α2-6 sialic acid to N-glycosylated proteins and the EGFR ligand, amphiregulin^[75]^. Interestingly, exomeres are enriched for bioactive miRNAs, which were formerly considered to be carried by exosomes [Table 2]^[76]^.

**Table 2:** Most abundant exomere and supermere proteins^[16,17]^

**Characteristics**	**Exomeres**	**Supermeres**
Sedimentation	196,000 *g* for 16 h	367,000 *g* for 16 h
Size	~35 nm	~29 nm
Associated with both	- HSP90 - PKM2 - FASN - LDHA - FLNB - EEF2/1A1	
Uniquely associated proteins (maximized at 10)	- ACTR3 - MAT1A - BZW1 - FAT4 - CALR - MTHFD1 - LGALS3BP - ERP44 - GPD1 - PFKL	- ENO1 - ACTN4 - HSP90AA1 - HSP90AB1 - HSPA8 - ALDOA - TKT - GPI

HSP90: Heat shock protein 90; PKM2: pyruvate kinase M2; FASN: fatty acid synthase; LDHA: lactate dehydrogenase A; FLNB: filamin B; EEF2/1A1: eukaryotic translation elongation factor 2/1A1; ACTR3: actin-related protein 3; MAT1A: methionine adenosyltransferase 1A; BZW1: basic leucine zipper and W2 domain-containing protein 1; FAT4: FAT atypical cadherin 4; CALR: calreticulin; MTHFD1: methylenetetrahydrofolate dehydrogenase 1; LGALS3BP: galectin-3-binding protein; ERP44: endoplasmic reticulum resident protein 44; GPD1: glycerol-3-phosphate dehydrogenase 1; PFKL: phosphofructokinase, liver type; ENO1: enolase 1; ACTN4: alpha-actinin-4; HSP90AA1: heat shock protein 90 alpha family class A member 1; HSP90AB1: heat shock protein 90 alpha family class B member 1; HSPA8: heat shock 70 kDa protein 8; ALDOA: aldolase A; TKT: transketolase; GPI: glucose-6-phosphate isomerase.

Supermeres are highly enriched with miR-1246 and miRNA-binding proteins, including argonaute proteins (AGO1 and AGO2), hnRNPA2B1, and exportin-5 (XPO5)^[17]^. They are capable of transferring intrinsic cellular characteristics; for example, exposure to supermeres derived from cetuximab-resistant cells results in cetuximab resistance in recipient cells^[17]^. The mechanisms involved in exomere and supermere biogenesis remain to be determined, and whether these mechanisms are exploited by cancer cells to modulate the tumor microenvironment requires further investigation [Table 2]^[16,17]^.

Overall, the considerable heterogeneity in EV composition and associated functions indicates several independent mechanisms for cargo sorting, depending on intrinsic cellular expression patterns, cell type, extrinsic factors, and cargo specificity.

### Degradation or secretion of EVs

**Figure 5:**
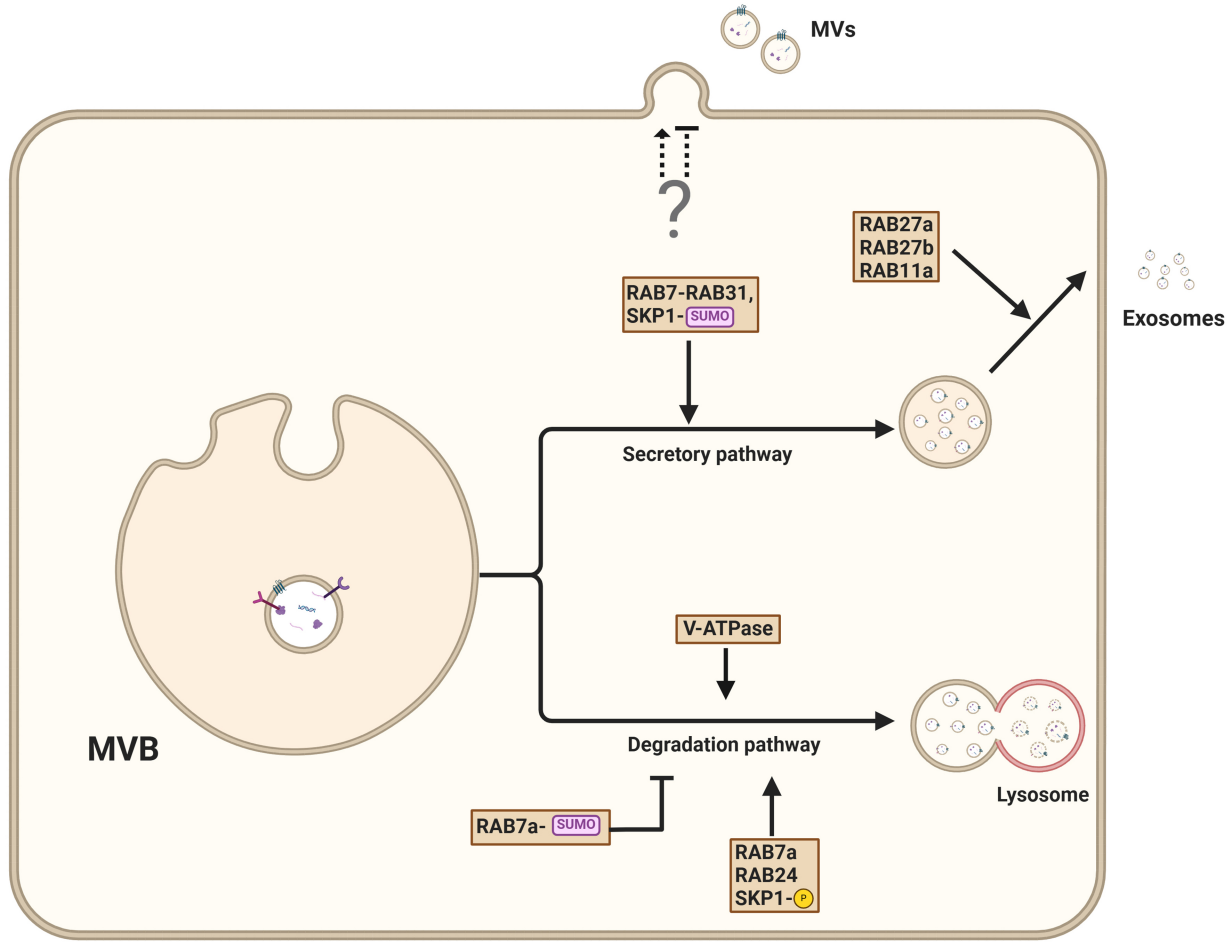
Pathways governing the secretion of exosomes from MVBs. MVBs can either fuse with the plasma membrane, resulting in the release of ILV as exosomes into the extracellular environment, or fuse with lysosomes for cargo degradation. The exosomal secretion pathway is facilitated by interactions that involve PSEN1, RAB27A, RAB31, and SUMOylated adapter protein SKP1, which promote vesicle trafficking to the plasma membrane. Conversely, lysosomal degradation is controlled by RAB7, RAB2A, and phosphorylated SKP1. The mechanism of microvesicle secretion remains poorly understood. Created in BioRender. Ju, J. (2025) https://BioRender.com/wcsvh0m. MVBs: Multivesicular bodies; ILVs: intraluminal vesicles; PSEN1: presenilin 1; SKP1: S-phase kinase-associated protein 1.

Although different sorting mechanisms exist, cargo sorted into MVBs may be subjected to lysosomal degradation^[21]^. Alternatively, degradation is circumvented and cargo is secreted via EVs [Figure 5].

EV secretion is closely linked to lysosomal status^[77,78]^. For example, preventing lysosomal acidification through V-ATPase inhibition enhances the release of EVs^[20]^. Interestingly, the impairment of lysosomal acidification through V-ATPase inhibition prevents autophagosome maturation and diverts autophagy intermediates to late endosomes. The resulting amphisomes enable RAB27A-dependent secretion of vesicles^[79]^. Similarly, preventing V-ATPase-mediated endosome acidification through nSMase2-mediated MVB membrane lipid composition alteration promotes the secretion of small EVs^[80]^.

Recent work has revealed that presenilins, particularly presenilin 1 (PSEN1), are important gatekeepers that control the balance between endosome release and their autophagy-dependent degradation^[81]^. PSEN deficiency results in decreased EV secretion due to autophagy hyperactivation, driving endosomes preferentially toward the lysosomal pathway instead of allowing their release^[81]^. Interestingly, V-ATPase inhibition using Bafilomycin A1 was found to restore EV secretion by stimulating unconventional secretion pathways.

The role of V-ATPase in secretory processes is further underscored by the α3 isoform, which is essential for lysosomal secretion through its regulation of RAB7 and RAB27A localization to lysosomes^[82]^.

RAB7 is a key regulator of late endosome and lysosome trafficking, playing a pivotal role in the secretion of cargo such as EVs, enzymes, and signaling molecules. Specifically, RAB7A is required for the EV-mediated release of miRNA through interaction with RAB27B^[83]^. Interestingly, the interaction of RAB7A with RAB24 and Rab-interacting lysosomal protein (RILP) results in vesicle degradation^[84]^, suggesting that post-translational control of RAB7A activity is decisive for vesicle fate. Although not entirely elucidated yet, different RAB7 subtypes or the (poly)ubiquitination status of RAB7A may influence its function; for example, preventing RAB7 ubiquitination results in enhanced EV secretion^[85]^. In addition to ubiquitination, post-translational modification of RAB7 by SUMOylation prevents the degradation of *Salmonella Typhimurium*^[86]^.

Further illustrating the important role of RAB proteins in fate decisions, RAB31 GTPase regulates exosome biogenesis independently of the traditional ESCRT and tetraspanin pathways^[87]^. RAB31 promotes ILV formation and blocks the fusion of MVBs with lysosomes, thereby enhancing exosome production. RAB31 overexpression is associated with increased exosome production and EGFR packaging^[87,88]^. The RAB31-dependent ILV formation is based on interactions with ceramide- and cholesterol-rich membranes and flotillin-1^[87,89]^. Additionally, RAB31 inhibits RAB7 activity at MVBs, blocking MVB-lysosome fusion and promoting EV release^[87]^.

The exocyst is a conserved octameric complex that coordinates the final steps of vesicle trafficking and fusion with the plasma membrane. It plays a crucial role in targeting vesicles to specific membrane sites, ensuring the precise and regulated release of vesicle content. RAB11a is required for the interaction between MVBs and the exocyst^[90]^. The coordinated binding of RAB11a and the exocyst at MVBs results in their delivery to the cell membrane, where they assemble with membrane-associated components EXO70 and SEC3. The full complex enables the fusion of MVBs with the plasma membrane^[90]^. In summary, these findings suggest that RAB proteins and post-translational modifications determine vesicle fate, directing them toward either the degradation or secretory pathway.

ISGylation, a post-translational modification that results in protein conjugation of ISG15 (interferon-stimulated gene 15), decreases the number of MVBs, and impairs exosome secretion^[45]^. ISG15 functions in immune responses and protein homeostasis, and its modification of target proteins can alter their stability and interactions. ISG15 conjugation triggers MVB co-localization with lysosomes and promotes the aggregation and lysosomal degradation of MVB. Accordingly, inhibition of lysosomal function or autophagy restores exosome secretion^[45]^. Specifically, ISGylation of the ESCRT-I core component TSG101 enhances its aggregation and subsequent degradation, thereby inhibiting EV secretion^[45]^.

ESCRT-independent associated proteins also influence the decision between degradation and secretion. For example, syntenin-1 is recruited to tetraspanin 6 (TSPAN6)-positive MVB. TSPAN6 inhibits autophagosome and lysosomal functions, increases the number of ILV, and results in enhanced secretion of EVs^[91]^. Other TSPANs, such as CD9, also positively affect the rate of exosome release^[92]^.

Interestingly, the release of EVs is dependent on the polarization of cells. On the apical side, the secretory pathway-associated (SSA) machinery governs the release of exosomes, while on the basolateral side, sphingomyelinase-dependent ceramide production contributes to the formation of exosomes^[93]^.

Post-translational modifications, such as ubiquitination, SUMOylation, and ISGylation, regulate EV sorting and secretion by modulating key proteins, including TSG101, RAB7a, and ESCRT components. RAB31 promotes exosome biogenesis by inhibiting MVB-lysosome fusion, while the exocyst complex and RAB11a direct MVB transport to the plasma membrane. Tetraspanins and ceramide-dependent mechanisms further modulate exosome release. These findings highlight the complex interplay between degradation and secretory pathways, emphasizing the need for future research to explore how these processes coordinate to regulate EV fate.

## INTERPLAY BETWEEN AUTOPHAGY AND EV BIOGENESIS AND SECRETION

Autophagy and EV biogenesis and release share regulatory mechanisms and molecular machineries. For instance, the post-translational modifications (such as Ubiquitylation, ISGylation, SUMOylation, NEDDylation, phosphorylation, and glycosylation) that control cargo sorting into EVs also control cargo segregation into autophagosomes^[12,45]^.

**Figure 6:**
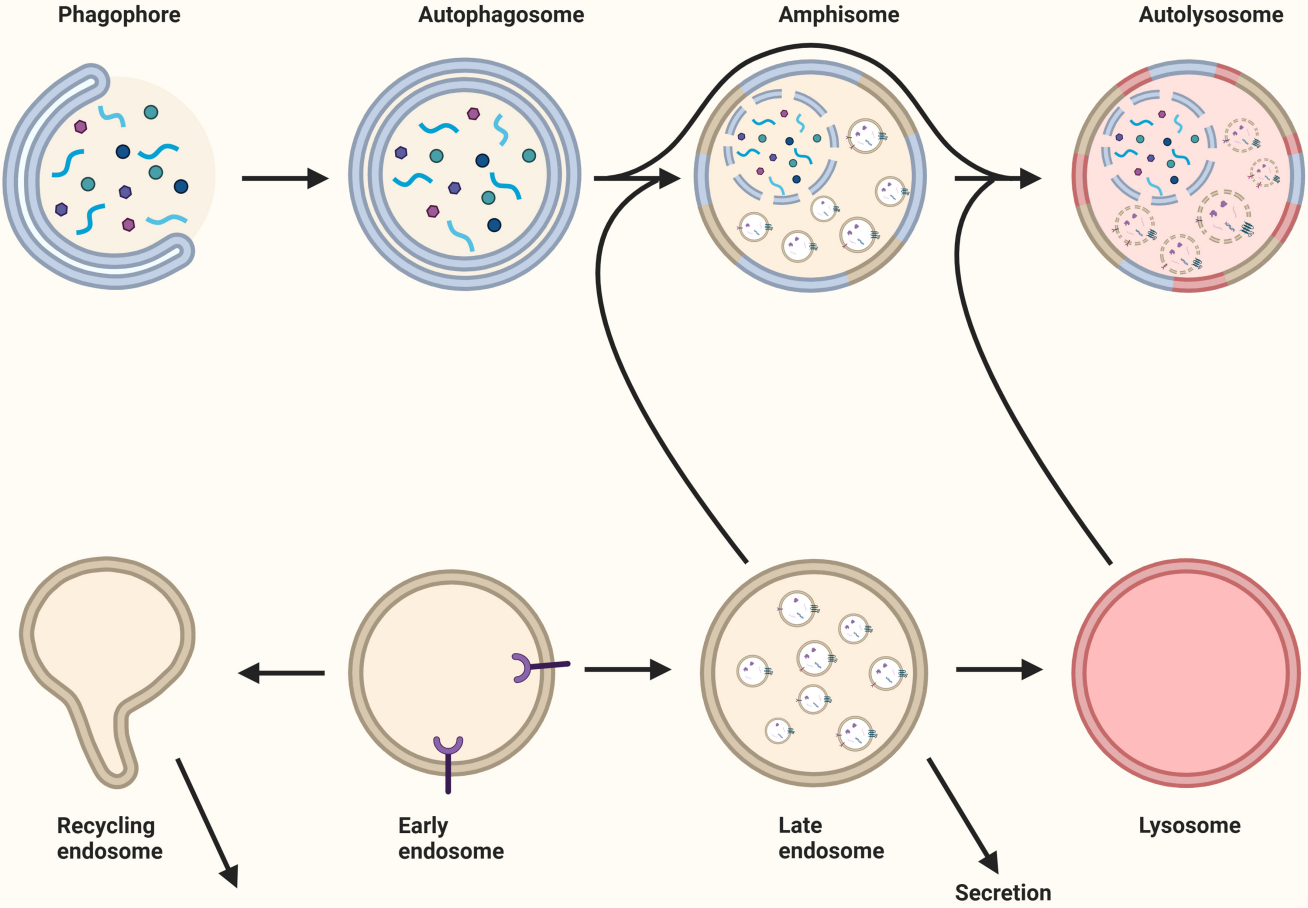
The relationship between autophagy and extracellular vesicles. Following the formation of the autophagosome through the elongation of the phagophore, the autophagosome fuses with the recycling endosome, late endosome, or lysosome to generate an amphisome. For the degradation of the cargo, the amphisome fuses with a lysosome to create an autolysosome. Late endosomes and recycling endosomes may fuse with the plasma membrane for secretion. Created in BioRender. Ju, J. (2025) https://BioRender.com/iq8sd3z.

Macroautophagy is the best-characterized pathway of autophagy^[94]^ and results in the degradation of intracellular components^[95]^. Cargo is degraded through fusion of the autophagosomes with lysosomes or endosomes, forming autolysosomes or amphisomes, respectively [Figure 6]^[96]^. CMA and microautophagy bypass autophagosome formation and directly target cargo to lysosomes. CMA and microautophagy bypass autophagosome formation and directly deliver cargo to lysosomes. Both processes are regulated in a stress-dependent manner and primarily involve heat shock protein family A (Hsp70) member 8 (HSPA8), which recognizes and targets substrates for degradation^[27,97,98]^. In CMA, HSPA8 binds to substrate proteins containing a KFERQ-like motif and facilitates their translocation across the lysosomal membrane. During microautophagy, HSPA8-bound substrates are delivered to lysosomes or endosomes via direct membrane invagination^[27,96]^. Endosomal microautophagy has been shown to resemble exosome biogenesis, as cargo is recruited by comparable ESCRT-dependent processes, while microautophagy is independent of ESCRT^[27,97]^.

Modulating canonical autophagy, either by stimulation or inhibition, influences EV secretion, highlighting potential cross-talk between these cellular pathways^[12,97]^. In addition, autophagy facilitates unconventional secretion (secretory autophagy), which involves the release of cytosolic proteins missing a N-terminal peptide (leaderless proteins) and therefore bypassing the conventional secretory pathway^[99]^.

### Autophagy and EV biogenesis

Basal autophagy activity is essential for maintaining cellular homeostasis during normal conditions. Yet, autophagy is rapidly induced during exposure to stressors, such as hypoxia and starvation^[100]^. The mechanistic target of rapamycin (mTOR) signaling pathway responds to nutrient availability and controls autophagy activity. In nutrient-rich conditions, mTOR phosphorylates the Unc-51-like autophagy activating kinase 1 (ULK1), thereby inhibiting autophagy. Conversely, during nutrient-deprived conditions, mTOR is suppressed, leading to ULK1 activation and autophagy induction^[12,101]^. Notably, mTOR inhibition not only induces autophagy but also stimulates the release of exosomes^[102]^ and highlights the functional connection between autophagy and EV release. Furthermore, many proteins involved in phagophore and autophagosome formation are also involved in EV biogenesis, underscoring a tight regulation and cross-talk between these seemingly distinctive mechanisms^[103]^.

**Figure 7:**
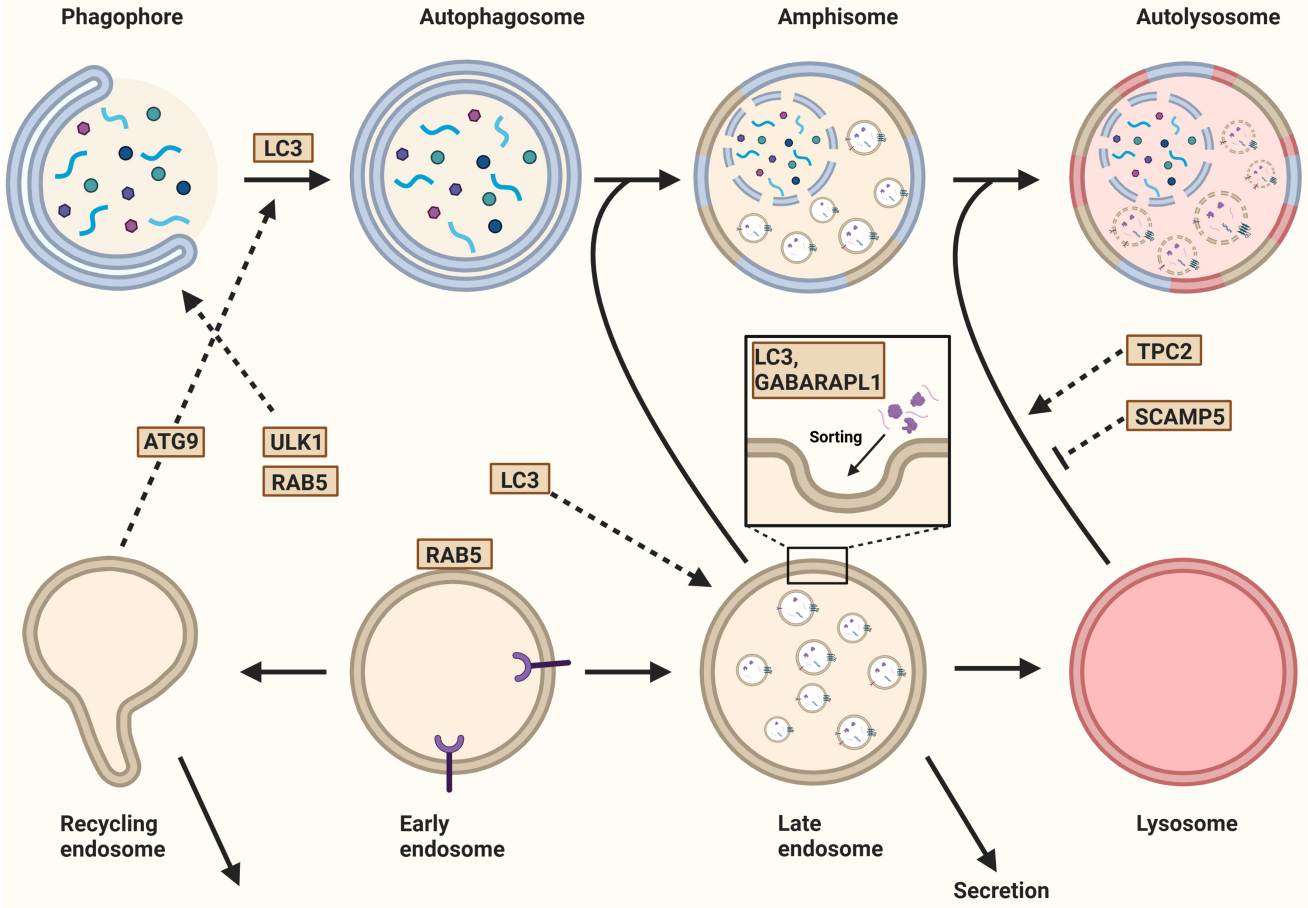
Autophagy and the biogenesis of extracellular vesicles. After the initiation of autophagy, ULK1 interacts with the endosomal protein RAB5, leading to nucleation. The recycling endosome contributes to this process by trafficking the ATG9 from the recycling endosome to the autophagosome. Additionally, LC3 is recruited to the expanding phagophore for membrane expansion and autophagosome formation. In eukaryotes, ATG8 has evolved into the LC3/GABARAP protein family, which includes six members such as LC3B and GABARAPL1. These proteins regulate both cargo loading and the secretion of extracellular vesicles. Once formed, the autophagosome either fuses with a MVB to generate an amphisome or with a lysosome to form an autolysosome. The fusion between the autophagosome and lysosome is regulated by TPC2, a lysosomal non-selective sodium/calcium channel. Similarly, SCAMP5 inhibits this fusion, thereby regulating cargo segregation. Finally, the fusion of late or recycling endosomes with the plasma membrane results in the secretion of their contents. Created in BioRender. Ju, J. (2025) https://BioRender.com/vnlxtuy. ULK1: Unc-51-like autophagy activating kinase 1; ATG9: autophagy-related gene 9; LC3: microtubule-associated protein 1 light chain 3; GABARAPL1: gamma-aminobutyric acid receptor-associated protein-like 1; MVB: multivesicular body; TPC2: two-pore channel 2; SCAMP5: secretory carrier-associated membrane protein 5.

For example, once autophagy is initiated, ULK1 is activated and interacts with the early endosomal protein RAB5, leading to the recruitment of Beclin 1 complex I. This complex, which consists of Class III phosphoinositide 3-kinase (PI3K) VPS34, VPS15, and autophagy-related protein ATG14L, facilitates the nucleation of the phagophore^[12,27,95]^. Alternatively, complex II is formed when VPS34 and VPS15 bind to UV radiation resistance-associated gene (UVRAG)^[104]^. The subsequent phosphorylation of PI(3)P into PIP_3_ by those complexes results in phagophore expansion. Activation of VPS34 can also be induced by the early endosomal protein RAB5, and the maturation of the RAB5 domain requires the VPS34-derived PI(3)P to deactivate RAB5. An essential step in EV biogenesis is the maturation of early endosomes into MVBs, which is driven by the replacement of RAB5 by RAB7. MVBs are required for sorting and secondary trafficking^[4,31]^. Interaction between RAB5 and VPS34 in altering phosphorylation status suggests that autophagy, endosomal maturation, and EV biogenesis are interlinked^[95,105]^ [Figure 7].

RE serve as a source for EV production and are central to cellular homeostasis by facilitating the retrieval of specific proteins and lipids from early endosomes back to the plasma membrane. This process is essential for maintaining the composition and functional properties of the plasma membrane^[80]^. Interestingly, RE contain ULK1 and ATG9, yet on different subdomains, suggesting specialized roles for these proteins during different cellular processes^[106]^. During starvation-induced autophagy, transferrin and its receptor are recruited to RE in a process mediated by ULK1, which is negatively regulated by the Rab GTPase-activating protein (RabGAP) TBC1D14. This results in the tubulation of RE, disrupting the recruitment of transferrin and its cognate receptor^[95]^. Together with the trafficking protein particle (TRAPP)-III, TBC1D14 controls the trafficking of ATG9. Overexpression of the binding region of TBC1D14 that interacts with TRAPPIII blocks both autophagy and secretory trafficking^[107]^, suggesting that ATG9 localization determines the fate of RE^[107]^.

Another RabGAP involved in both autophagy and endosomal trafficking is TBC1D5^[95]^. In the absence of TBC1D5 or retromer (an endosomal multi-protein complex that organizes the endocytic recycling of a vast range of integral membrane proteins), the activity and localization of RAB7 become dysregulated. Instead of being restricted to late endosomes, lysosomes, the endoplasmic reticulum, the TGN, and mitochondrial membranes, hyperactivated RAB7 expands over the entire lysosomal domain. This lysosomal accumulation of hyperactivated RAB7 results in a striking loss of RAB7 mobility and overall depletion of the inactive RAB7 pool on endomembranes^[108]^. Moreover, this regulation of RAB7 has been observed to facilitate the sorting of ATG9A and the generation of autophagosomes during Parkin-mediated mitophagy^[108]^ [Figure 7]. The importance of endosomes in EV biogenesis and their interaction with autophagy-associated proteins emphasizes the interplay between autophagy and EV biogenesis.

Phagophore expansion and autophagosome formation require microtubule-associated protein 1 light chain 3B (MAP1LC3B) integration. This is tightly controlled by ATG7-ATG3-mediated phosphatidylethanolamine conjugation and ATG5-ATG12-mediated membrane elongation^[101,109]^ [Figure 7]. In addition to its function in autophagy, ATG5 decreases the acidification of late endosomes, which is required for the movement and maturation of organelles involved in the endosomal-lysosomal pathway. By preventing the fusion of MVBs with lysosomes, ATG5 promotes the release of exosomes. Gou *et al.* suggested that this decrease in acidification is mediated by ATG5 by disassociating the vacuolar proton pump V_1_V_0_-ATPase from the MVB^[110]^, indicating that autophagy-related molecules directly control EV biogenesis^[110]^. Beyond the classical ATG12-ATG5 and ATG3-ATG7 complexes, ATG12 can also covalently bind to ATG3. The ATG12-ATG3 complex results in the accumulation of late endosomes and impaired basal autophagic flux. Interestingly, this complex recruits ALIX, an ESCRT-associated protein, which contributes to exosome biogenesis and the distribution of late endosomes^[111]^.

Further interactions between autophagy and EV biogenesis are exemplified by members of the LC3/GABARAP protein family (microtubule-associated protein 1 light chain 3 (LC3) isoforms A, B, and C, as well as gamma-aminobutyric acid receptor-associated protein (GABARAP), GABARAP-like 1 (GABARAPL1), and GABARAPL2^[112]^. For example, proteomic analyses have identified various proteins and non-coding RNAs, particularly small nucleolar RNAs (snoRNAs) and miRNAs, whose release depends on LC3^[113]^ [Figure 7]. Leidal *et al.* established that the two RNA-binding proteins (RBPs), hnRNP-K and scaffold-attachment factor B (SAFB), are released within EVs after interacting, possibly via their LIR motifs, with LC3^[114]^. Moreover, Gardner *et al.* confirmed transferrin receptor (TFRC) loading into EVs through LIR motifs^[115]^.

GABARAPL1 is involved in intracellular transport, including the delivery of EGFR to the plasma membrane^[116]^. It is also enriched in MVBs and is essential for endosome maturation, cargo sorting into endosomes, and EV secretion in a RAB7-dependent manner. Additionally, GABARAPL1 is present on EV membranes and marks a subset of EVs with pro-angiogenic^[117]^ and pro-metastatic properties^[118]^.

In addition to the direct involvement of autophagy-related proteins in EV biogenesis, inhibition of late-stage autophagy can alter the EV secretome. For example, aggregation of MAPT/Tau disrupts the ANP32-A-INHAT-IST1-ESCRT-III pathway, inhibiting autophagosome-lysosome fusion^[119]^. Concurrently, inhibition of this pathway increases the loading of -synuclein into EVs^[77]^. Similarly, secretory carrier membrane protein 5 (SCAMP5) blocks lysosome-autophagosome fusion while increasing the secretion of -synuclein via exosomes^[120]^ [Figure 7]. These findings suggest that SCAMP5 coordinates autophagy and exosome biogenesis to efficiently clear toxic proteins through their loading into exosomes rather than by autophagy.

In summary, autophagy and EV biogenesis are intricately linked processes that share regulatory pathways, molecular machinery, and functional outcomes. Both rely on endosomal trafficking and involve key proteins such as RAB5, RAB7, and GABARAPL1, which play critical roles in cargo selection, vesicle formation, and transport. Autophagy-related proteins, including ATG5 and LC3, not only govern autophagosome formation but also influence EV biogenesis by modulating endosome maturation, exosome production, and cargo loading.

Post-translational modifications, including LC3 conjugation, further highlight the molecular overlap between these pathways, affecting both autophagic flux and vesicle secretion. Moreover, disruptions in autophagy, such as lysosomal dysfunction or inhibition of autophagosome-lysosome fusion, often lead to compensatory EV release, suggesting a functional interplay in maintaining cellular homeostasis and stress responses.

### Autophagy and the degradation and secretory pathways of EVs

#### Regulation of EV degradation and secretion through autophagy

Several studies have demonstrated that autophagy and EV secretion are interconnected. For example, Zou *et al.* observed that EV release is regulated by mTORC1, a master coordinator of autophagy. Additionally, CD63 deficiency led to increased autophagy, as indicated by enhanced LC3 conversion^[102,121]^. This increase in autophagy was accompanied by a reduction in EV secretion, suggesting a shift toward cargo degradation rather than secretion.

Conversely, PIKfyve regulates autophagy by ensuring proper lysosomal function, autophagosome-lysosome fusion, and cargo degradation. Dysfunction of PIKfyve disrupts autophagic flux and lysosomal homeostasis. Inhibition of PIKfyve has been shown to increase EV secretion, highlighting the complex role of these proteins in EV dynamics^[122]^.

Moreover, inhibition of autophagosome formation has been shown to promote EV release. For instance, the prion protein inhibits autophagosome formation by interfering with the inhibitory effects of caveolin^[123]^.

**Figure 8:**
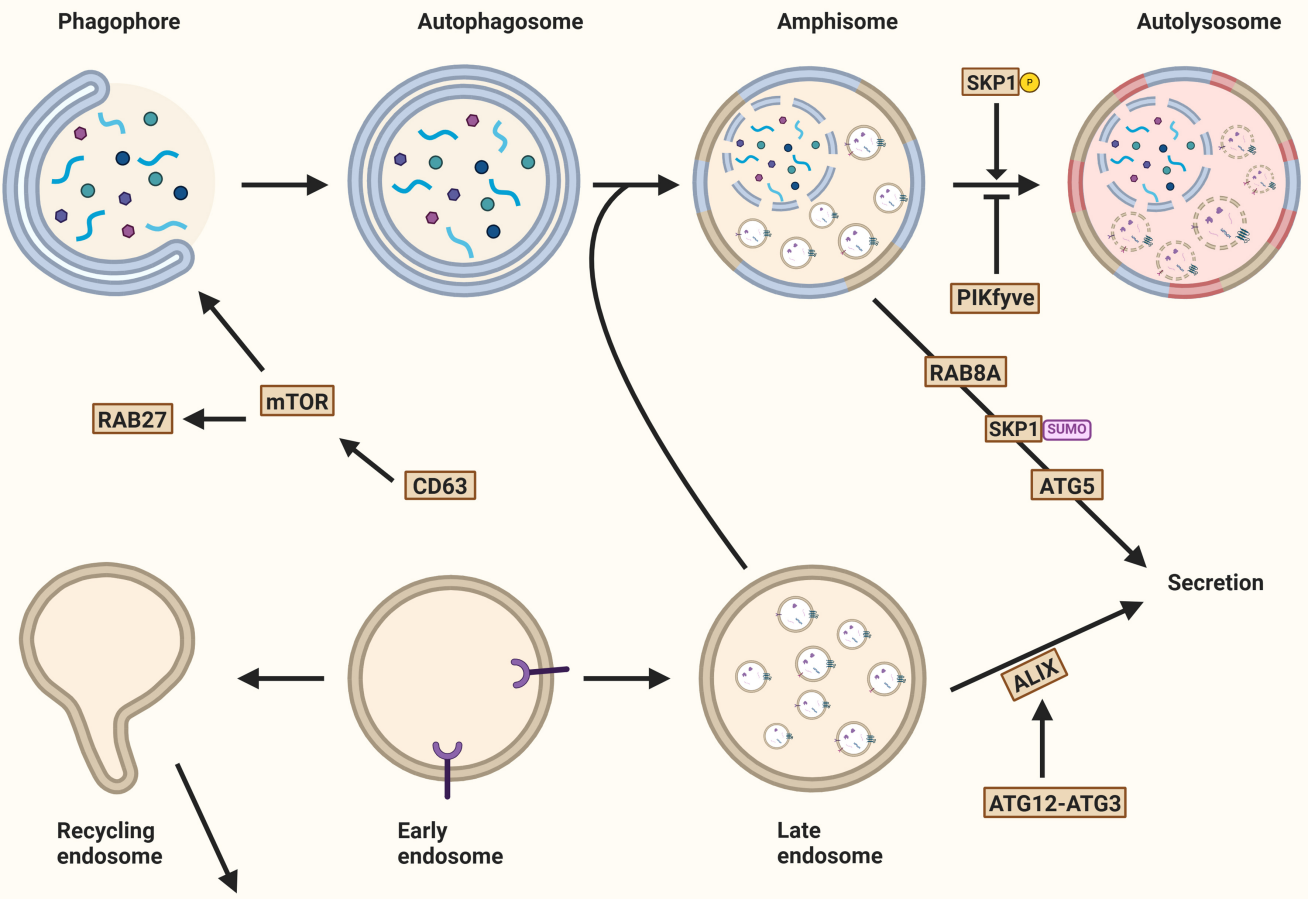
Autophagy and the secretion of extracellular vesicles. Several mechanisms determine whether autophagosomal and endosomal cargo is degraded or released into the extracellular environment. Key regulators include mTOR, a master autophagy modulator, and tetraspanin CD63, whose knockout activates mTOR. PIKfyve kinase activity promotes phagophore expansion and EV secretion. The interaction of ATG12 and ATG3 also promotes EV secretion through ALIX recruitment. Secretory autophagy, an unconventional secretory pathway, depends on ATG5 and RAB8. Additionally, RAB27A and the inhibition of PIKfyve contribute to secretory autophagy. Created in BioRender. Ju, J. (2025) https://BioRender.com/qoefza9. mTOR: Mechanistic target of rapamycin; EV: extracellular vesicle; ATG12: autophagy-related gene 12; ALIX: ALG2-interacting protein X; SKP1: S-phase kinase-associated protein 1.

Although the role of ESCRT proteins in EV biogenesis is well established, Leidal *et al*. concluded that the secretion of RBPs and LC3 is independent of the ESCRT machinery but rather relies on the autophagy machinery. This includes neutral sphingomyelinase SMPD3, which produces ceramide during EV biogenesis, and the LC3-dependent recruitment of its regulator, factor associated with nSMase2 activity FAN/NSMAF^[114]^ [Figures 7 and 8].

These observations, along with evidence that fusion of MVBs and autophagosomes typically leads to cargo degradation, suggest that autophagy machinery is largely involved in determining whether cargoes follow the degradation or secretory pathway, or that multiple EV subclasses are dependent on distinct biogenesis pathways, which requires further investigation.

#### Secretory autophagy

Proteins that lack a leader signal are not secreted through the conventional ER-Golgi pathway, but instead follow unconventional routes, such as secretory autophagy^[124]^.

One of the earliest identified examples of secretory autophagy is the release of interleukin (IL)-1. As a leaderless protein, IL-1 is synthesized as an inactive precursor that resides in the cytosol. Upon activation by the inflammasome - a cytosolic multi-protein complex involved in innate immunity - IL-1β is processed and subsequently packaged into vesicles for secretion. This process involves the autophagy-related protein ATG5 and the small GTPase RAB8A, both of which are critical for the non-canonical trafficking of IL-1β^[27,125]^.

Further mechanistic insights come from work by Zhang *et al.*, who demonstrated in yeast that mature IL-1 is translocated into the intermembrane space of autophagosomes with the aid of HSP90^[126]^. This study also showed that IL-1 is sorted into EVs in a TSG101-dependent manner, indicating that components of the ESCRT machinery may be involved. Completion of IL-1 secretion requires MVB formation and subsequent fusion with the plasma membrane^[50,126]^. However, the exact mechanisms by which IL-1 escapes degradation remain to be further elucidated.

Secretory autophagy is not limited to IL-1. Other leaderless proteins have also been shown to follow similar routes. For example, annexin A2 (ANXA2), a calcium-regulated membrane-binding protein, is secreted via an autophagy-dependent mechanism regulated by RAB8A and RAB27A, but not by RAB27B^[127]^. Knockout of ATG5 or expression of a mutant form of RAB11 reduces ANXA2 secretion, further supporting the involvement of the autophagic machinery in this pathway^[127]^.

Transforming growth factor beta 1 (TGF-1) is another leaderless cytokine that plays key roles in cell growth, proliferation, and differentiation. In fibroblasts, TGF-1 is selected by GORASP2 in the Golgi and secreted via secretory autophagosomes through a RAB8A-dependent pathway^[128]^. Notably, TGF-1 secretion is completely abolished in cells lacking key components of the autophagy machinery.

The glycolytic enzyme glyceraldehyde-3-phosphate dehydrogenase (GAPDH) is also released through an autophagy-related but macroautophagy-independent pathway. Instead, during microautophagy, GAPDH is incorporated into late endosomes, which then fuse with the plasma membrane to release GAPDH via exosomal and lysosomal exocytosis^[129]^.

RASAL2, a Ras GTPase-activating protein (RasGAP), has emerged as a negative regulator of secretory autophagy. Silencing RASAL2 enhances exosomal release and increases the presence of autophagy-related proteins in EVs, suggesting a role in modulating the balance between degradation and secretion^[130]^.

Secretory autophagy and EV release are initiated by inhibition of the phosphoinositide kinase PIKfyve^[122]^. PIKfyve inhibition leads to an increase in MVBs, ILVs, and secreted EVs. Interestingly, the EV fraction from PIKfyve-deficient cells is enriched in autophagosomal proteins such as LC3B-II and selective cargo receptors including p62. These findings suggest that impaired lysosome-autophagosome fusion caused by PIKfyve inhibition diverts autophagic cargo toward secretion. This compensatory secretion likely helps maintain cellular proteostasis under conditions of disrupted degradation^[122]^.

In line with this, Solvik *et al.* observed that inhibition of lysosomal acidification significantly increases the secretion of autophagy cargo receptors such as p62/SQSTM1, NBR1, OPTN, and NDP52 through EVs and particles (EVPs), a term encompassing all membrane-bound vesicles and non-membranous particles^[79]^. These cargo receptors are primarily associated with small EVPs rather than being encapsulated within EVs. The small GTPase RAB27A plays a critical role in this secretion process, as RAB27A-deficient cells display reduced secretion^[79,115]^. Additionally, when maturation into autolysosomes is impaired, the secretion of autophagy cargo receptors via EVPs helps maintain the balance of these proteins within the cell, thereby mitigating intracellular protein accumulation^[12,79,131]^.

In addition to EVs, emerging evidence suggests that other extracellular nanoparticles, such as exomeres and supermeres, may also participate in autophagy-related secretion. Although mechanistically distinct from classical vesicular trafficking, these particles are enriched in proteins involved in stress responses, metabolism, and proteostasis - processes closely linked to autophagy. Moreover, their cargo often includes leaderless proteins and molecular chaperones, hinting at a role for unconventional secretion pathways, including autophagy. These observations suggest that exomeres and supermeres may serve as complementary or alternative routes for the disposal of autophagy-regulated proteins, especially under conditions of lysosomal dysfunction or cellular stress.

Proteomic analyses of exomeres and supermeres have revealed a significant enrichment of proteins such as HSP90, PKM, FASN, and LDHA, all of which play key roles in cellular stress responses, metabolism, and signaling. For instance, HSP90, a molecular chaperone, stabilizes autophagy-related proteins such as ULK1 and facilitates CMA^[132]^. FASN, a key enzyme in lipid synthesis, influences lipophagy by regulating lipid droplet dynamics and maintaining metabolic homeostasis^[133]^. LDHA, which catalyzes the conversion between pyruvate and lactate, affects autophagy through metabolic reprogramming. LDHA inhibition triggers autophagy under energy stress conditions^[134]^. Notably, HSP90, LDHA, and FASN lack classical leader signal peptides, suggesting that their secretion may depend on unconventional mechanisms, such as autophagy.

Interestingly, supermeres are enriched with HSPA8^[16,17]^, a protein that labels cargo for CMA-dependent degradation via ESCRT-independent recruitment^[27,97,98]^. While the exact mechanisms governing the secretion of exomeres and supermeres remain to be fully elucidated, the presence of leaderless proteins and chaperones in these complexes suggests a functional relationship with autophagy.

Overall, MVBs and autophagosomes can release their cargo through distinct molecular mechanisms that regulate the decision between cargo degradation or secretion [Table 3]. Although several studies distinguish between EV secretion and secretory autophagy, no standard criteria currently exist to clearly differentiate these processes. Consequently, it remains unclear whether these processes are truly distinct, interconnected, or simply different terminologies describing a single pathway. Moreover, the enrichment of autophagy-related proteins and chaperones in exomeres and supermeres suggests a role in autophagy-dependent secretion, or secretory autophagy. Further research is needed to specify the mechanisms and molecules involved in the decision-making process and to determine the extent to which these pathways are interlinked between the autophagic and endosomal systems.

**Table 3:** Characteristics distinguishing degradative autophagy from secretory autophagy

**Characteristics**	**Degradative autophagy**	**Secretory autophagy**
MVB fusion	Variable	Yes
Destination of autophagosome	Lysosome (degradation)	Plasma membrane (secretion)
Positive regulatory proteins	- SKP1-Phosphorylation	- RAB8A - RAB27A - SKP1-SUMOylation - ATG5 - RAB11 - TPC2
Negative regulatory proteins	- SCAMP5 - PIKfyve - RAB27A - TPC2	

MVB: Multivesicular body; SKP1: S-phase kinase-associated protein 1; RAB8A: Ras-related protein 8A; RAB27A: Ras-related protein 27A; SUMO: small ubiquitin-related modifier; ATG5: autophagy-related gene 5; RAB11: Ras-related protein 11; TPC2: two pore channel 2; SCAMP5: secretory carrier membrane protein 5.

## CONCLUSION

The sorting of diverse cargo, including proteins, lipids, and RNAs, into EVs is orchestrated by highly coordinated mechanisms. Key players in EV biogenesis include lipids, RAB proteins, TSPANs, and the ESCRT machinery. Subunits of the ESCRT complex work in conjunction with ALIX, which recognizes specific post-translational modifications critical for cargo selection.

Beyond their roles in cargo sorting, RABs and components of the ESCRT machinery are also vital for the final release of EVs. Over the past decade, research has shown that EV biogenesis extends beyond traditional ESCRT-dependent pathways, incorporating non-ESCRT mechanisms and auxiliary factors, such as ceramide-enriched lipid microdomains and other scaffolding proteins, to facilitate cargo loading and vesicle formation.

This expanding understanding of EV generation underscores the complex interplay among multiple pathways and regulatory factors, reflecting the versatility of EV biogenesis in cellular communication and homeostasis. ESCRT-independent mechanisms, such as those mediated by tetraspanins, lipid rafts, and small GTPases, play significant roles in the generation and sorting of cargo into EVs, particularly in the production of plasma membrane-derived EVs. Additionally, beyond the mutual recruitment of ESCRT proteins to form complexes, individual core ESCRT proteins can independently participate in EV generation. Auxiliary factors such as VPS4, ATPase, ALIX, and syntenin-1 are also critical for EV formation. Additionally, the dynamic distribution of membrane lipids, particularly lipid microdomains and membrane curvature, is essential for driving membrane budding. The mechanisms of EV generation are further influenced by cell type and physiological conditions, including stress, inflammation, and cell death.

Emerging evidence suggests that the current definitions of ESCRT-dependent and -independent sorting mechanisms should be broadened to include a more diverse array of protein and lipid participants, as well as the influence of cellular environments and conditions on EV biogenesis. This expanded framework reflects the complex and adaptable nature of EV formation.

There is also significant interplay between EV biogenesis, release, and autophagy. Autophagy relies on endosomal components and their trafficking for vesicle formation and also regulates cargo loading and EV release. Although both MVBs and autophagosomes recruit cargo, the molecular pathways determining whether cargo follows a degradation or secretory route are likely distinct, highlighting the specialized roles of these cellular systems.

In conclusion, EV biogenesis is a multifaceted process regulated by a complex interplay of proteins, lipids, and auxiliary factors. Traditional ESCRT-dependent mechanisms, along with emerging ESCRT-independent pathways, demonstrate the adaptability and diversity of cargo sorting and vesicle formation. These processes are further influenced by cellular contexts, including physiological conditions and autophagic pathways. The substantial overlap in molecular pathways, shared proteins, and complementary mechanisms indicates a high level of coordination between autophagy and EV biogenesis. Therefore, modulating one mechanism - whether through inhibition or enhancement - is likely to trigger compensatory responses from other pathways. While the observed biological effects may remain significant, it is important to consider these interactions when interpreting experimental results.

## DECLARATIONS

### Copyright

© The Author(s) 2025.
